# Application background and mechanism of short-chain fatty acids in sepsis-associated encephalopathy

**DOI:** 10.3389/fcimb.2023.1137161

**Published:** 2023-03-28

**Authors:** Qiulei Zhang, Chang Lu, Weixuan Fan, Jingxiao Zhang, Yongjie Yin

**Affiliations:** ^1^ Department of Emergency and Critical Care, The Second Hospital of Jilin University, Changchun, China; ^2^ Department of Anesthesiology, The Second Hospital of Jilin University, Changchun, China

**Keywords:** short-chain fatty acids, sepsis-associated encephalopathy, gut-microbiota-brain-axis, neuroinflammation, microglia, dietary intervention

## Abstract

Sepsis-associated encephalopathy (SAE) is a frequent brain dysfunction found in sepsis patients, manifesting as delirium, cognitive impairment, and abnormal behaviors. The gut microbiome and short-chain fatty acids (SCFAs) are particularly associated with neuroinflammation in patients with SAE, thus noticeably attracting scholars’ attention. The association of brain function with the gut-microbiota-brain axis was frequently reported. Although the occurrence, development, and therapeutic strategies of SAE have been extensively studied, SAE remains a critical factor in determining the long-term prognosis of sepsis and is typically associated with high mortality. This review concentrated on the interaction of SCFAs with microglia in the central nervous system and discussed the anti-inflammatory and immunomodulatory effects of SCFAs by binding to free fatty acid receptors or acting as histone deacetylase inhibitors. Finally, the prospects of dietary intervention using SCFAs as dietary nutrients in improving the prognosis of SAE were reviewed.

## Introduction

Sepsis is a life-threatening organ dysfunction caused by a dysregulated host response to infection (Singer et al., 2016). The central nervous system (CNS) is mainly the first to be affected in the course of sepsis, and it typically manifests as cognitive dysfunction, also known as sepsis-associated encephalopathy (SAE), which has a major role in the poor long-term prognosis of septic patients (Feng et al., 2019). Unfortunately, sepsis is still incurable, leading to high morbidity and mortality rates despite a broad range of therapeutic strategies, such as fluid resuscitation, enhanced screening, routine microbiological cultures for pathogenic factors, broad-spectrum antimicrobial therapy, etc (Rhodes et al., 2017). Consequently, it is necessary to improve the therapeutic strategies. Notably, sepsis leads to alterations in gut microbial diversity and abundance and a reduction in short-chain fatty acids (SCFAs), which may be associated with cognitive dysfunction in septic survivors (Wu et al., 2020; Giridharan et al., 2022; Yuan et al., 2022). Therefore, whether SCFAs are beneficial to treating neurological dysfunction in SAE has noticeably attracted researchers’ attention.

SCFAs are the primary end-products of the fermentation of non-digestible carbohydrates. The most abundant SCFAs in the colon and feces include acetate, propionate, and butyrate (Louis and Flint, 2017). In recent years, numerous studies have extensively investigated SCFAs in nerve injury. Butyrate administration facilitates the differentiation of oligodendrocytes, contributes to the suppression of demyelination, and enhances remyelination in the cuprizone-induced demyelination mice model (Chen et al., 2019). SCFAs ameliorate experimental autoimmune encephalomyelitis by promoting the polarization of naive T cells to regulatory T (Treg) cells and suppressing the p38 and JNK1 pathways. This manifests as reduced inflammatory cell infiltration, attenuated demyelination, and increased axonal preservation (Haghikia et al., 2015). Sadler et al. confirmed that the connection with the cerebral cortex induced by SCFAs leads to changes in the density of neurons in the spinal cord and synapses, significantly improving limb motor function recovery in stroke models (Sadler et al., 2020). In addition, SCFAs can effectively induce the transformation of microglia from M1 pro-inflammatory phenotype to M2 anti-inflammatory phenotype, thus reducing the inflammatory response after nerve injury, promoting the repair of the blood-brain barrier (BBB), and recovering the cerebral function in mice with depressive-like behaviors (Tang et al., 2022). Notably, SCFAs also influence neuroinflammation in the CNS by affecting glial cell morphology and function, thus providing a basis for subsequent research.

Herein, we reviewed the application background and mechanism of SCFAs as dietary nutrients in SAE to elucidate possible associations with the gut-microbiota-brain axis and provide more favorable evidence for the application of SCFAs in the field of SAE therapy.

## Application background of SCFAs in sepsis

SCFAs are the end products of the fermentation of dietary fibers by the anaerobic intestinal microbiota. Dietary fibers affect the human microbiome’s metabolism and change the host’s health status (Tanes et al., 2021). In sepsis, gut microbiota disturbance may reduce the concentrations of various components of SCFAs in feces and blood, which in turn induces cognitive impairment associated with the increased number of GFAP-positive cells in the prefrontal cortex and hippocampus (Giridharan et al., 2022). Also, in sepsis, the pathogenic colonization decreases the concentrations of SCFAs at 6 weeks after hospitalization (Yamada et al., 2015). Therefore, we summarized the background and underlying mechanisms to provide a theoretical basis for applying SCFAs in sepsis.

Partial G protein-coupled receptors (GPRs) located on the surface of cells act as free fatty acid receptors (FFARs) that have an important role in disease regulation. In particular, FFAR2 (GPR43) and FFAR3 (GPR41) are activated by SCFAs (Kimura et al., 2020), while exogenous administration of SCFAs (acetate: propionate: butyrate at a ratio of 3: 1: 1) can effectively alleviate CLP -induced decrease levels of acetic acid and propionic acid in SAE mice. They can also significantly increase the relative abundance of SCFAs-producing bacteria such as Allobaculum. SCFAs are critical in alleviating neuroinflammation and improving cognitive dysfunction in SAE patients through FFAR2 (Liao et al., 2022). Moreover, the intestinal flora and its metabolite butyrate have an important role in host SAE susceptibility. Especially the antioxidant stress and neuroprotective effects of butyrate associated with SCFA receptor GPR109A contribute to ameliorating long-term cognitive impairment in SAE (Zhang et al., 2022). The studies above supported that binding SCFAs to receptors is essential in improving neuroinflammation and cognitive dysfunction. Meanwhile, studies that administered SCFAs before the onset of sepsis did not fully confirm the effectiveness of SCFAs treatment. However, the underlying mechanism is not only associated with these receptors, as intracellular targets associated with histone deacetylases (HDACs) also have a relevant role. HDACs are of essential importance in the modification of chromosome structure and gene expression regulation. SCFAs are natural inhibitors of HDACs. Sodium butyrate, as a histone deacetylase inhibitor (HDACi), may reverse aversive memory in septic animals by reducing HDAC activity after cecal ligation and puncture (CLP) surgery (Steckert et al., 2015). In addition to the administration of SCFAs at different times and different routes, the study of indole-3-propionic acid (IPA) as a microbiota metabolite of tryptophan (an essential amino acid derived from dietary supplementation), which contribute to modulating gut dysbiosis in septic mice also illustrates the role of the gut microbiome and its metabolites in the pathophysiology of sepsis through different pathways (Fang et al., 2022). In addition, butyrate may enhance synaptic plasticity and improve depressive-like behaviors and cognitive performance by inhibiting the histone deacetylase (Citraro et al., 2020; Yu et al., 2020). Collectively, the administration of SCFAs as HDACi contributes to a new treatment strategy for managing neurological dysfunction with cognitive impairment.

In addition, SCFAs can exert anti-inflammatory, antioxidant, and immunomodulatory effects. Exogenous administration of SCFAs enhances macrophage function, ameliorates *K. pneumoniae*-induced pulmonary inflammation, and improves pneumonia sepsis symptoms (Wu et al., 2020). Administration of sodium butyrate after the onset of sepsis can reduce proinflammatory cytokine production, attenuate intestinal injury and improve survival rate by inhibiting nuclear factor-κB (NF-κB) activation in CLP-induced septic rats, which suggests that clinical application of sodium butyrate may potentially contribute to inhibition of systemic inflammatory response and sequential organ dysfunction (Fu et al., 2019). Meanwhile, pretreatment with butyrate attenuated the elevation of pro-inflammatory mediators such as TNF-α, IL-6 and IL-1β levels in LPS–induced septic mice or in murine macrophage-like RAW 264.7 cells, which were stimulated by LPS. Moreover, significantly upregulating the expression of anti-inflammatory IL-10 is an important way butyrate alleviates the inflammatory response of sepsis (Wang et al., 2017). These studies suggested that SCFAs could improve the severity of sepsis and may even increase survival rate through anti-inflammatory pathways. Filippone et al. confirmed sodium propionate’s anti-inflammatory and antioxidant effects (Filippone et al., 2020). However, whether SCFAs have an active anti-inflammatory role depends on their concentrations, serum pH, and etiologies (Tedelind et al., 2007). Furthermore, in their clinical study, Weng et al. reported that propionate levels were well correlated with sepsis severity and prognostic information, which is of great significance for the next follow-up research (Weng et al., 2018) (**Table 1**). Meanwhile, previous studies also confirmed significantly lower concentrations of stool SCFAs in clinical septic patients (Valdés-Duque et al., 2020). Moreover, ten days after sepsis induction, the animals still suffered cognitive impairment associated with decreased SCFAs levels, which were triggered by disruption of microbiota-gut-brain axis homeostasis (Giridharan et al., 2022). Accordingly, the treatment with SCFAs to maintain the concentration of SCFAs, especially after the onset of sepsis, may be more indicative of the reliability of SCFAs in critically ill patients with sepsis. However, future studies are needed to address this issue further. Additionally, when SCFAs are taken up into T lymphocytes, SCFAs-derived acetyl groups contribute to the increase of cellular acetyl-CoA, which may influence the histone acetylation and cytokine gene expression, including the promotion of IL-10 production (Sun et al., 2018; Luu and Visekruna, 2019). However, SCFAs have been found to drive the differentiation of naive CD4^+^ T cells into Treg cells and supply a viable target for treating autoimmune diseases (Bhutia and Ganapathy, 2015; Asarat et al., 2016). These findings confirmed the anti-inflammatory, antioxidant, and immunomodulatory effects of SCFAs. The role of SCFAs in energy metabolism is noteworthy. Previous studies have demonstrated that SCFAs can be used as substrates to participate in fat, cholesterol and glucose metabolism to regulate cellular energy metabolism (den Besten et al., 2013; Perry et al., 2016). The microglial mitochondrial functional deficiencies that are rectified by acetate, an SCFA, lead to improved microglial metabolism and the shaped innate immune mechanism during neurodegeneration (Erny et al., 2021). In summary, these findings better clarify the research background of SCFAs related to sepsis and lay the foundation for further research on the mechanism of application of SCFAs in SAE.

**Table 1:** Overview of the application background of SCFAs to sepsis models.

Reference	Species	Modeling method	Measurement of SCFAs	Conclusion
(Giridharan et al., 2022)	Rat	CLP	feces, 16S rRNA	The gut microbiota, SCFAs, and glial cell may contribute to the treatment of cognitive impairment in septic survivors.
(Wu et al., 2020)	Mouse	Intranasal inoculate with K. pneumoniae	cecal contents and serum, GC-MS	SCFAs ameliorate *K. pneumoniae*-induced pulmonary inflammation.
(Liao et al., 2022)	Mouse	CLP	Feces, GC-MS	SCFAs protect the cognitive function in SAE mice via GPR43.
(Zhang et al., 2022)	Mouse	CLP	Feces, GC	Butyrate, as a metabolite of the gut microbiota, may affect the susceptibility of mice to SAE.
(Steckert et al., 2015)	Rat	CLP	ND	Sodium butyrate administration may contribute to reverse the cognitive damage in septic survivors.
(Fu et al., 2019)	Rat	CLP	ND	Sodium butyrate can mitigate the inflammatory response and maintain intestinal barrier function in polymicrobial sepsis.
(Wang et al., 2017)	Mouse	LPS	ND	Butyrate, a SCFA, can significantly attenuate the inflammation against sepsis.
(Filippone et al., 2020)	J774-A1 cell line	LPS	ND	Propionate, a SCFA, exhibits anti-inflammatory effects on LPS-induced inflammation via significantly reducing NF-κB translocation.
(Weng et al., 2018)	Human	Severe sepsis	Blood, GC-MS	Serum propionic acid serves as a significant predictor and a prognostic biomarker in septic patients.

CLP, cecal ligation and puncture; GC-MS, gas chromatography-mass spectrometer; GPR, G protein-coupled receptor; GC, gas chromatography; ND, not done; HDACi, histone deacetylase inhibitor; LPS, lipopolysaccharide.

## Mechanism of SCFAs applied to SAE

**Figure 1:**
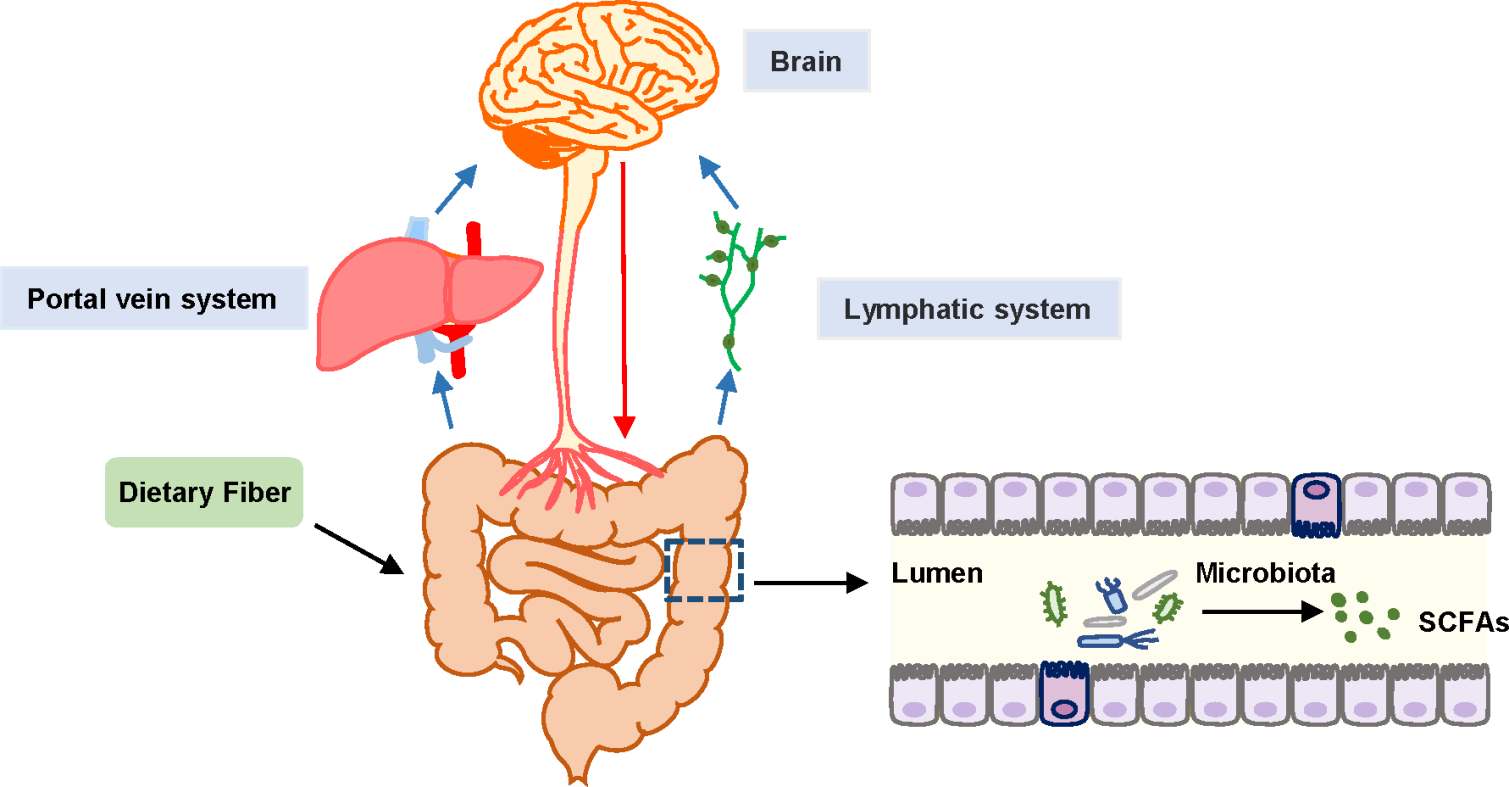
Schematic diagram of gut-microbiota-brain axis. Black arrows represent SCFAs that are the end products of fermentation of dietary fibers metabolized by the gut microbiome. Blue arrows represent the transport of SCFAs from the gut to distant organs through the portal vein or to the thoracic duct and subsequently to the blood through the mesenteric lymph nodes, and ultimately transfer to the blood. Red arrows represent the association between the enteric nervous system (ENS) and CNS, contributing to the regulation of brain function.

The gut microbiota influences the neuroendocrine system by regulating endocrine signals that enteroendocrine cells (EECs) produce. Then, the cerebral function may be further regulated through the correlation between the enteric nervous system (ENS) and CNS (Rao and Gershon, 2018). The gut microbiota and some metabolites can be translocated from the gut to distant organs through the portal vein or into the thoracic duct through mesenteric lymph nodes, and may ultimately transfer to the blood, influencing the brain (Doig et al., 1998; Deitch, 2012) (**Figure 1**). However, the increase of pathogenic microbiota in the gut and the decrease of gut microbiota products, such as SCFAs, can aggravate brain disorders such as anxiety, pain, depression, autism, Alzheimer’s disease, Parkinson’s disease, etc (Nagpal et al., 2019; Sharon et al., 2019; Zhu et al., 2020). In sepsis, this may be followed by SAE. The pathophysiology of SAE comprises neuroinflammation and microglial activation, BBB dysfunction, mitochondrial dysfunction, neurotransmitter dysfunction, etc (Mazeraud et al., 2020). Previous research indicated that the mechanisms related to SCFAs and SAEs might involve the following aspects.

### Neuroinflammation and immunity regulation

Neuroinflammation is the main mechanism underlying the development of SAE (Nardelli et al., 2016). SCFAs (acetate: propionate: butyrate at a ratio of 3: 1: 1) can significantly reverse behavioral impairment, such as a decrease in reflex and sensory function, neuropsychiatric state, and motor behavior in the SAE mice. They can also significantly increase the levels of ZO-1 and occludin associated with BBB integrity and significantly inhibit neuroinflammation by suppressing the JNK and NF-kB signaling pathways in CLP-induced SAE models (Liu et al., 2021). In addition, acetic acid and propionic acid, as SCFAs, reduce the expression levels of pro-inflammatory cytokines, such as IL-1β, IL-6, and tumor necrosis factor-α (TNF-α) through the NF-κB pathway (Guo et al., 2021). SCFAs also increase the expression level of the anti-inflammatory factor IL-10 (Wang et al., 2017). However, Li et al. suggested that SCFAs can ameliorate hippocampal neuroinflammation by activating the colonic NLRP6 inflammasome independently of peroxisome proliferator-activated receptor-γ (PPAR-γ) activation and increasing DCX^+^ newborn neurons in the hippocampus (Li et al., 2019). Therefore, SCFAs could improve neurological disorders by inhibiting neuroinflammation.

Microglia may act as innate immune cells in the brain, which participate in the secretion of cytokines (e.g., IL-1β, IL-6, and TNF-a). Therefore, they are significant in the occurrence and development of neurological and psychiatric diseases (Wendeln et al., 2018; Li et al., 2020). Several studies have identified the mechanism of SCFAs on host immunity, in which the binding of SCFAs to FFAR has an important role. FFAR2 and FFAR3 were reported to be associated with intracellular Ca^2+^ release, inhibition of cyclic adenosine monophosphate (cAMP) accumulation, mitogen-activated protein kinase (MAPK) and extracellular signal-regulated kinase ½ (ERK1/2) activation, thus contributing to modulation of immune and inflammatory responses (Le Poul et al., 2003). Although FFAR2 is not directly expressed in microglia, FFAR2 has an important role in the transformation of macrophages, especially in the transformation of the microglia to the anti-inflammatory M2 phenotype macrophages (Erny et al., 2015; Nakajima et al., 2017). In summary, SCFAs have a protective effect on the immune system. The binding of SCFAs can achieve these effects on FFARs to alter the macrophage phenotype, thereby reducing the release of proinflammatory factors.

### BBB

To the best of our knowledge, BBB serves as the main barrier contributing to CNS protection and maintaining brain homeostasis. The disruption of BBB leads to the activation of microglial cells and the secretion of pro-inflammatory cytokines, which may further aggravate brain damage in septic patients (Danielski et al., 2018). Braniste et al. suggested that SCFAs or metabolites produced by bacteria may affect BBB permeability by increasing the expression level of occludin in the frontal cortex and hippocampus (Braniste et al., 2014). In addition, acetic acid and propionic acid can improve the destruction of BBB by increasing the expression levels of tight junction (TJ) proteins in the hippocampus and alleviating cognitive dysfunction (Luo et al., 2021). However, propionate, an SCFA, exhibited protective effects on the BBB against oxidative stress by nuclear factor-erythroid 2 p45-related factor 2 (NRF2, also known as Nfe2l2) signaling pathway (Hoyles et al., 2018). Meanwhile, butyrate stabilizes hypoxia-inducing factor-1 (HIF-1), which protects BBB function (Kelly et al., 2015). Furthermore, M1-phenotype microglia under stress may induce a large amount of inducible nitric oxide synthase (INOS), in which INOS-mediated large production of NO may lead to metabolic hypoxia, gradually advancing to mitochondrial oxidative phosphorylation disorders. Subsequently, the increased production of reactive oxygen species (ROS) can induce apoptosis and aggravate neurological damage, which is also associated with further disruption of BBB (Zhao et al., 2019; Moraes et al., 2021). Taken together, SCFAs may regulate the expression levels of TJ proteins at the BBB and coordinate with microglia and mitochondria to regulate the BBB function and the development of neuroinflammation.

### Energy metabolism

**Figure 2:**
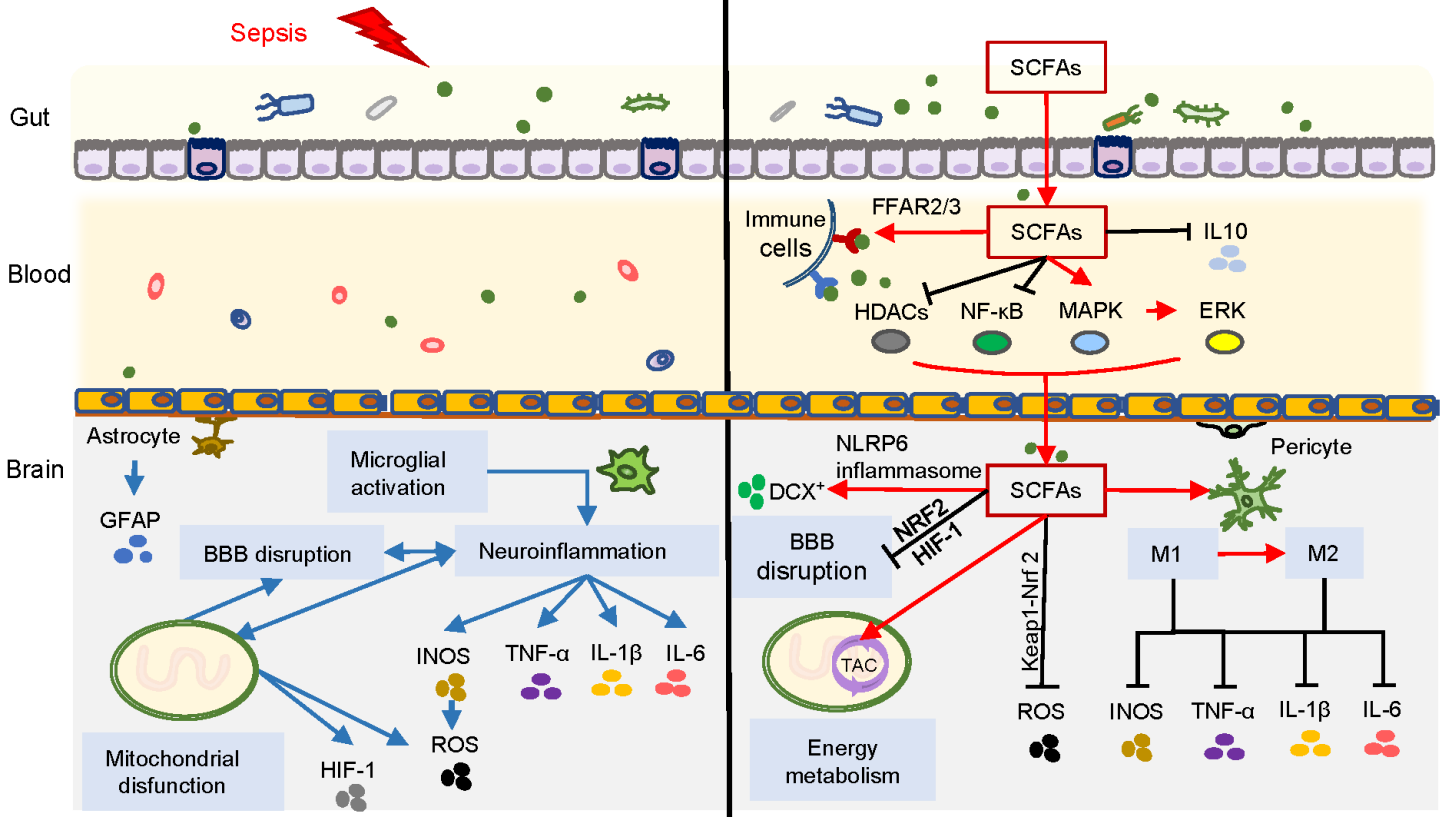
Overview of SCFAs correlated with SAE. Blue arrows on the left side of the figure represent the possible pathogenesis of SAE, involving neuroinflammation caused by microglial activation, as well as BBB damage and mitochondrial dysfunction. The right side of the figure represents the inhibition of neuroinflammation, immunomodulation, improvement of BBB function and energy metabolism exerted by SCFAs through different pathways during SAE (red arrows represent promotion, and black lines represent inhibition).

SCFAs are used as substrates for mitochondrial oxidation and the tricarboxylic acid cycle (TAC), and SCFAs may present positive effects on body weight control by regulating energy intake and energy expenditure. During sepsis, intra-mitochondria biogenesis is affected by oxidative stress, resulting in adaptive changes in intracellular glycolysis, oxidative phosphorylation (OXPHOS), and energy production (Sun et al., 2019). The alternative glycolytic pathways generate a large amount of ROS during adenosine triphosphate (ATP) production, leading to subsequent cell damage and even cell death. Furthermore, excessive ROS generation increases the production of HIF-1α and promotes the release of IL-1β associated with pro-inflammatory response (Mills et al., 2016). However, SCFAs, such as butyrate and propionate, are activators of the Kelch-like ECH-associated protein 1-nuclear erythroid 2-related factor 2 (Keap1-Nrf2) antioxidant defense pathway, which contributes to maintaining cellular redox homeostasis. Butyrate treatment can particularly reduce inflammatory markers and ROS production (González-Bosch et al., 2021). Moreover, gut microbiome-derived butyrate can modulate mitochondrial activity and enhance mitochondrial function at the gene level in the context of increased ROS production, which is associated with cognition function (Rose et al., 2018). The administration of SCFAs may improve mitochondrial function associated with energy metabolism, ameliorate inflammatory response, and exert an antioxidant role against oxidative stress during sepsis. Therefore, SCFAs, as signaling and key energy molecules, contribute to improving neuroinflammation, stabilizing immunomodulation and BBB function, and improving energy metabolism (**Figure 2**). However, whether these mechanisms are directly related to the prognosis of SAE needs to be further explored.

## Future prospects

The effects of alterations in microbiome and SCFAs on brain function were previously confirmed, providing a potential target for dietary intervention of SAE (Wu et al., 2021). However, it is essential to balance the predictable risk factors, such as transmitting infectious agents to new recipients caused by fecal microbiota transplantation (FMT) (Blaser, 2019). The remaining challenges can be summarized as follows: (1) it remains unclear what dietary nutrients can be used as a source of SCFAs for SAE remission, and what are the qualitative and quantitative criteria for the species and abundance of the gut microbiota associated with the metabolism of SCFAs in SAE; (2) although there is a close correlation between SCFAs and changes in pro-inflammatory and anti-inflammatory cytokines at mRNA levels during the inflammatory response to sepsis (Wang et al., 2017; Fu et al., 2019; Wu et al., 2020), the effects of SCFAs on gene expression in brain cells require further research; (3) further studies are needed to establish the exact relationship between different components of SCFAs and brain energy metabolism and energy acquisition in patients with SAE; and whether overdose and toxicity are involved. Solving the problems mentioned above and elucidating the exact mechanism may be remarkably beneficial for the prognosis of SAE patients.

## Conclusion

SCFAs may be involved in developing SAE by regulating neuroinflammation, immunity, BBB function, energy metabolism, etc., through multiple pathways. More importantly, SCFAs act on the CNS through the gut-microbiota-brain axis and exert some neuropsychological interventional effects. Therefore, alteration of the components of SCFAs *via* direct administration or dietary interventions may be a promising approach. In the future, the effects of different constituents of SCFAs on SAE should be studied in the context of gut microbiota to reveal the exact mechanism and improve the prognosis of SAE.

## Author contributions

QZ: contributed to the conceptualization, project administration, and preparation of the primary draft of the manuscript. CL and WF: contributed to the investigation and curation of resources and data. JZ and YY: contributed to supervision, funding acquisition, and review of the manuscript. All authors read and approved the final manuscript.
